# Supplementary material for: Novel Disulfiram-Loaded Metal–Organic Nanoparticles Inhibit Tumor Growth and Induce Immunogenic Cell Death of Triple-Negative Breast Cancer Cells
Source: Pharmaceutics. 2025 Nov 9;17(11):1448. doi: 10.3390/pharmaceutics17111448 (PMC12655402; doi:10.3390/pharmaceutics17111448)
Supplement: Supplementary file 1 [file pharmaceutics-17-01448-s001.zip › pharmaceutics-3885788-supplementary.pdf]

pharmaceutics

MDPI

Supporting Information

Novel Disulfiram-Loaded Metal–Organic Nanoparticles Inhibit Tumor Growth and Induce Immunogenic Cell Death of Triple-Negative Breast Cancer Cells

Chung-Hui Huang [1,†], Xuejia Kang [2,†], Lang Zhou [2], Junwei Wang [1], Shuai Wu [2], Peizhen Sun [2], Qi Wang [1], Adam B. Keeton [1], Pengyu Chen [2,*] and Gary A. Piazza [1,*]

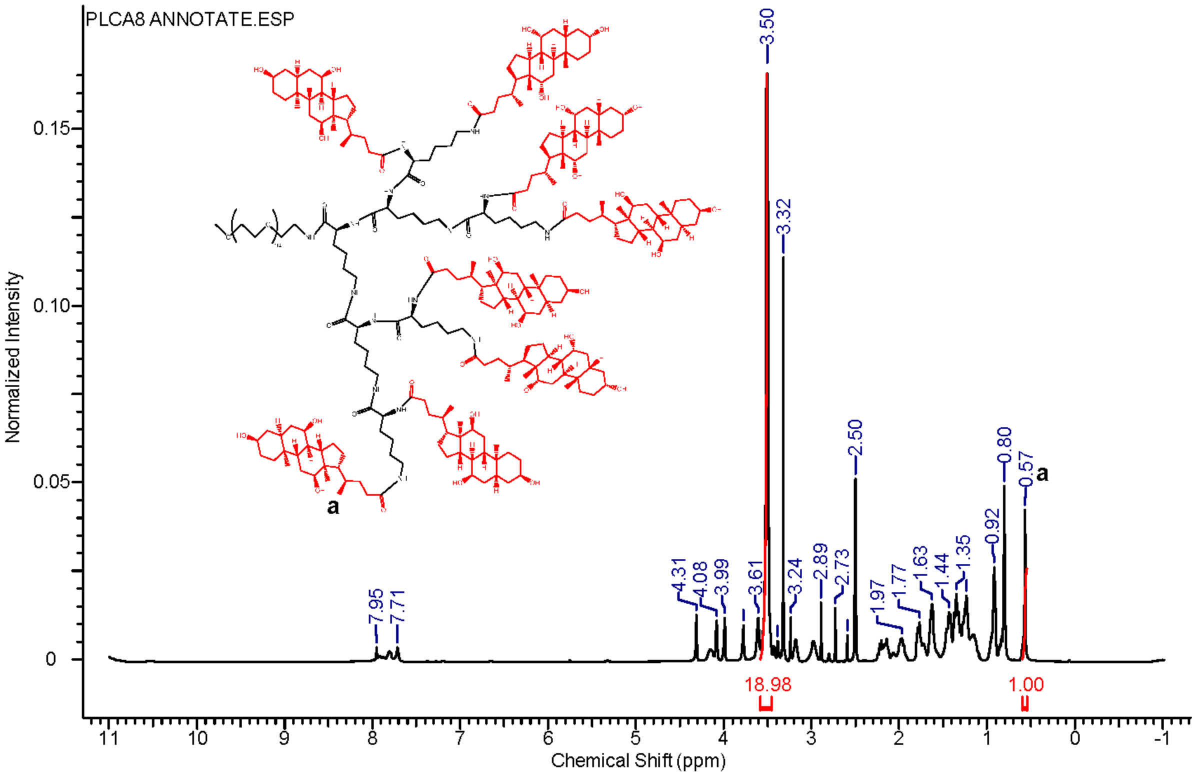

Figure S1. 1H NMR spectrum of mPEG-Lys4-CA8 in DMSO-d6. The degree of cholic acid substitution on the $mPEG_{5000}$-Lys_4 dendrimer was determined by quantitative analysis of the 1H NMR spectrum (DMSO-d_6). The broad resonance of the PEG backbone (δ 3.45–3.6 ppm) was integrated as 18.98, which corresponds to ~454 protons based on the theoretical composition of $mPEG_{5000}$. A characteristic singlet from a cholic acid methyl group (δ ~0.57 ppm, 3H) was integrated as 1.00. Normalizing for the number of protons, the substitution ratio was calculated as [(1/3)/(18.98/454)] = 7.97, and the substitution rate was (7.97/8) ×100% = 99.63%, which corresponds to ~8 cholic acid units per PEG-Lys4 macromolecule. This value confirms nearly complete substitution.

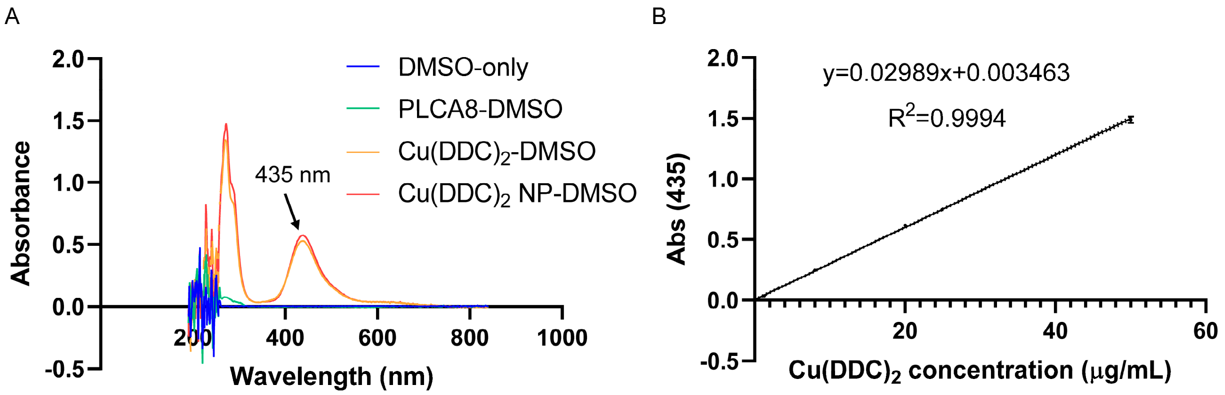

Figure S2. UV–Vis analysis and calibration of [$Cu(DDC)_2$] quantification. (A) Absorbance spectra of [$Cu(DDC)_2$], [$Cu(DDC)_2$] NPs, PLCA8 polymer, and DMSO control, confirming negligible interference from the polymer matrix at 435 nm. (B) Standard calibration curve of [$Cu(DDC)_2$] in DMSO (y = 0.02989x + 0.003463, R^2 = 0.9994).

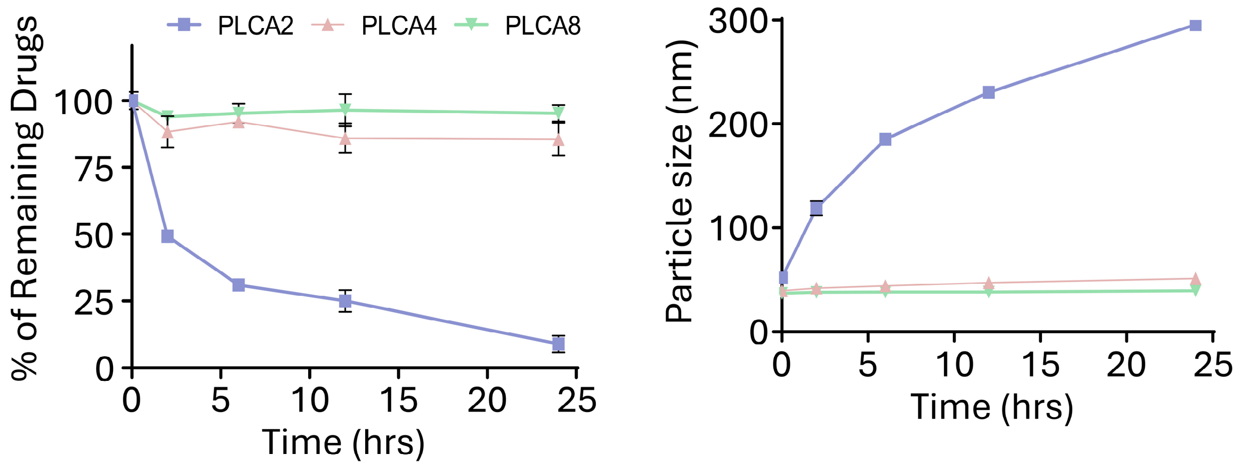

Figure S3. The performance of PLCA-formed NP. Left: Change in particle size of the NP during a 24-hours stability test. Right: Change in concentration of the NP during the stability test.

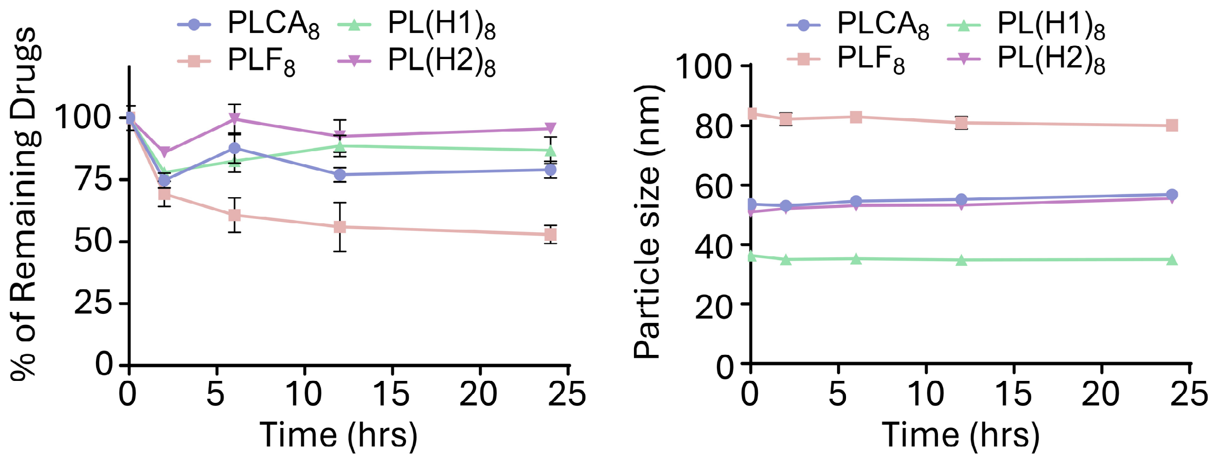

Figure S4. The performance of NP prepared with different PEG-lysine dendrimer derivatives. Left: Change in particle size of the NP during a 24-hours stability test. Right: Change in concentration of the NP during a 24-hours stability test.

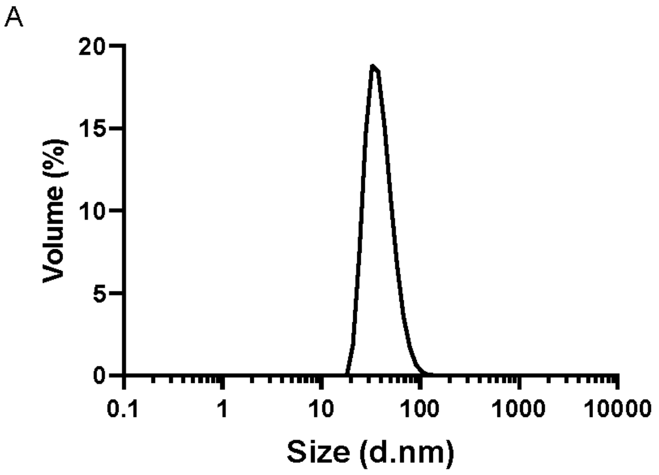

B

	Average size	PDI	Zeta potential
$Cu(DDC)_2$ NPs	46 ± 0.353 nm	0.116 ± 0.012	-3.12 ± 1.24 mv

Figure S5. DLS characterization of [$Cu(DDC)_2$] NP. (A) The particle size distribution showing a single, narrow peak centered at ~46 nm. (B) Summary of DLS measurements: average size, polydispersity index (PDI), and zeta potential (mean ± SD, n = 3 technical replicates).

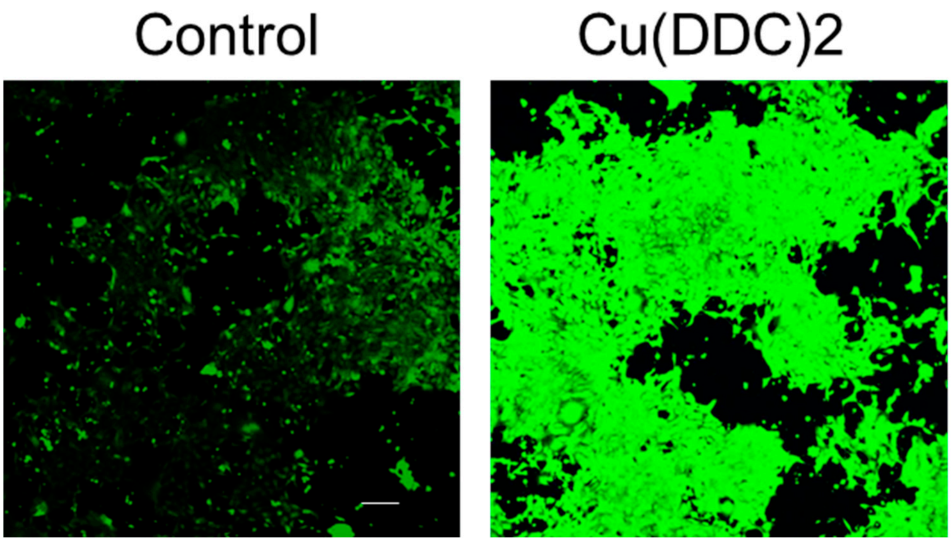

Figure S6. Detection of ROS generation in 4T1 cells using DCFDA staining, scale bar= 100 µm.

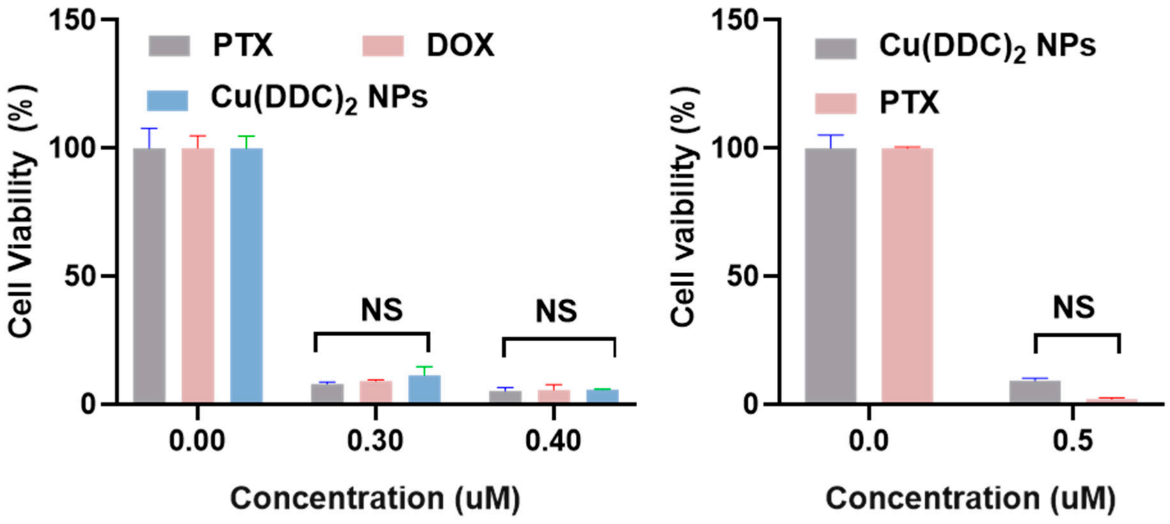

Figure S7. The cell viability of 4T1 in response to [Cu(DDC)2], DOX, PTX. NS, no significance (one-way ANOVA).

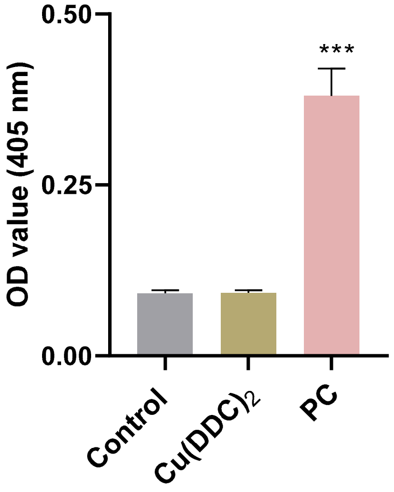

Figure S8. Caspase-3 after 4 h treatment: [Cu(DDC)2] NP 0.2 μM, and Staurosporine 1 μM (positive control). Mean ± SD (n = 3) ***P < 0.001 vs. control (one-way ANOVA, Dunnett).

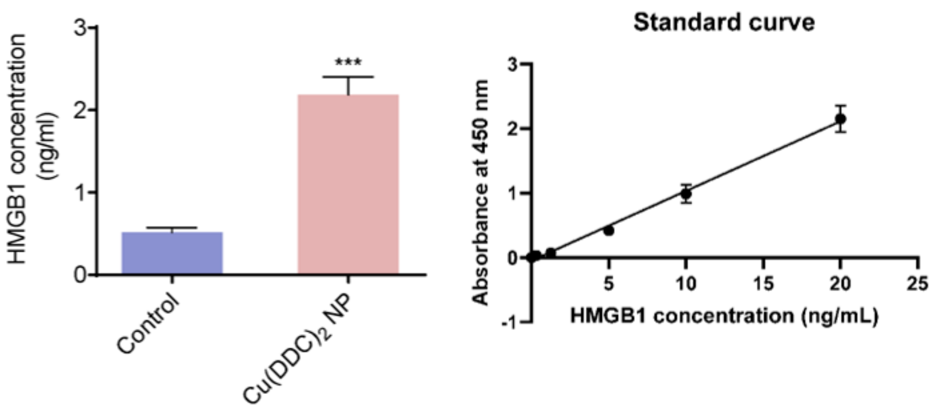

Figure S9. Quantification of HMGB1 release from 4T1 cells with or without [Cu(DDC)2] NP treatment. (n = 3, ***, P<0.001) Left: ELISA quantification of extracellular HMGB1 concentration in 4T1 cells following treatment with [Cu(DDC)2] NP compared to untreated group. Right: Standard curve

of the HMGB1 ELISA kit generated by plotting absorbance at 450 nm against HMGB1 concentration (ng/mL).

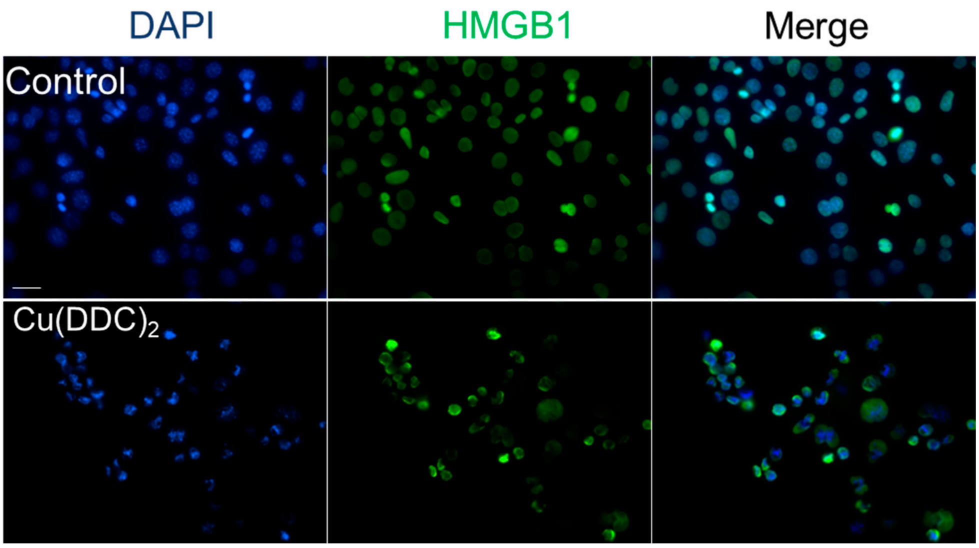

Figure S10. Intracellular localization of HMGB1 in cancer cells with or without [Cu(DDC)$_2$] NP treatment. Scale bar = 50 μm.

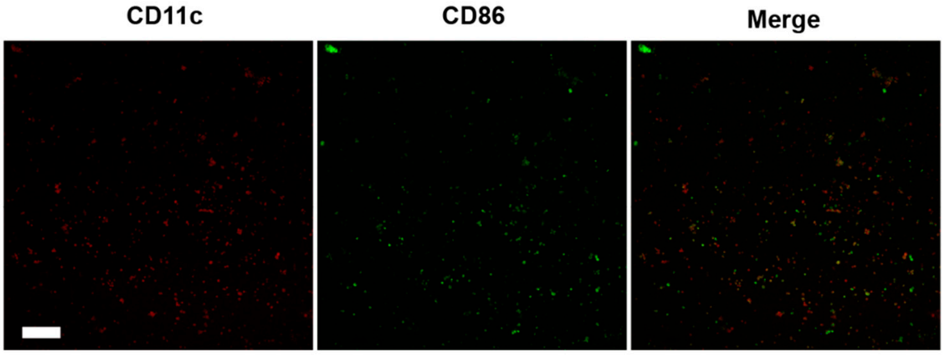

Figure S11. DCs maturation in response to [Cu(DDC)$_2$]-treated cancer cells. DCs maturation was assessed by CD86 expression following exposure to cancer cells pretreated with [Cu(DDC)$_2$] NP. Increased CD86 levels indicate enhanced DCs activation, suggesting immunogenic effects of the nanoparticle treatment. Representative immunofluorescence images are shown. Scale bar = 100 μm.

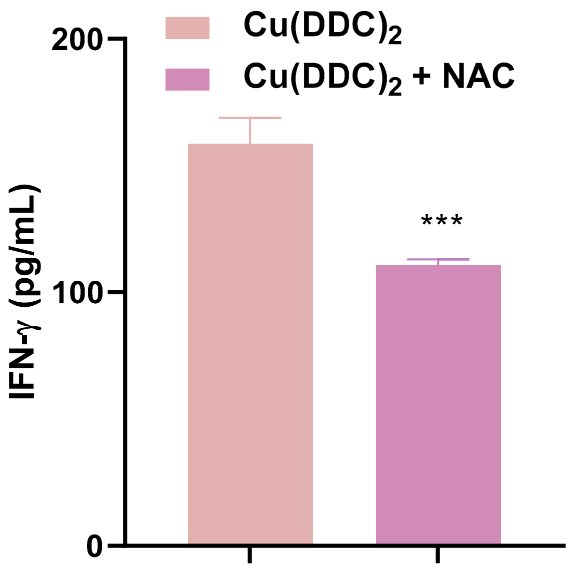

Figure S12. Effect of ROS scavenging on IFN-γ release in the tumor microenvironment. Statistical analysis was performed using an unpaired t-test between control and [Cu(DDC)₂] NP-treated groups (***P < 0.001).

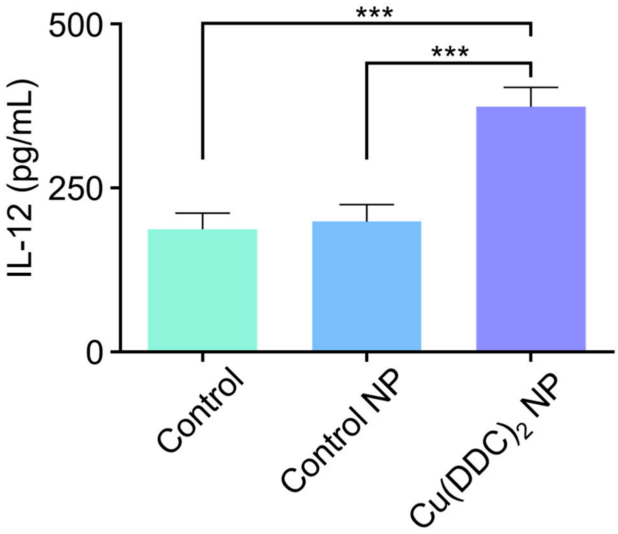

Figure S13. IL12 levels in the co-culture of tumor cell, DC and T cells exposed to [Cu(DDC)₂] NP. Unpair t test between control and Cu(DDC)₂ NP ***, P<0.001.

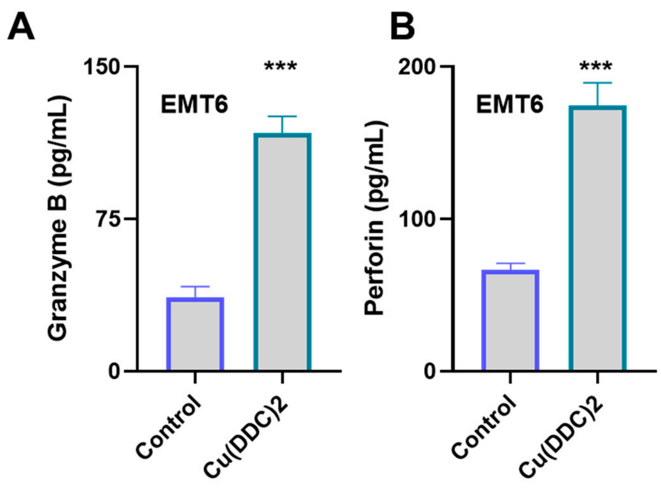

Figure S14. Secretion of cytotoxic factors in tumor/DC/T cell co-culture treated with [Cu(DDC)$_2$] NP. Unpair t test between control and [Cu(DDC)$_2$] NP ***, P<0.001.

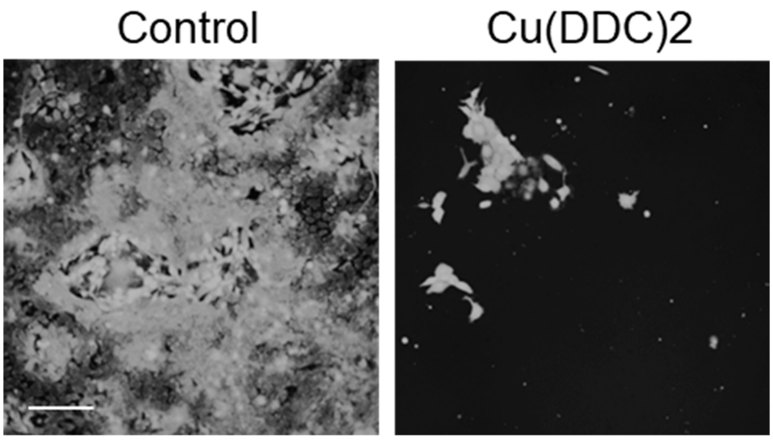

Figure S15. Cell migration at 48 h. Left, vehicle control; right, [Cu(DDC)$_2$] (48 h) with visibly reduced migrated cells. Scale bar, 100 μm.

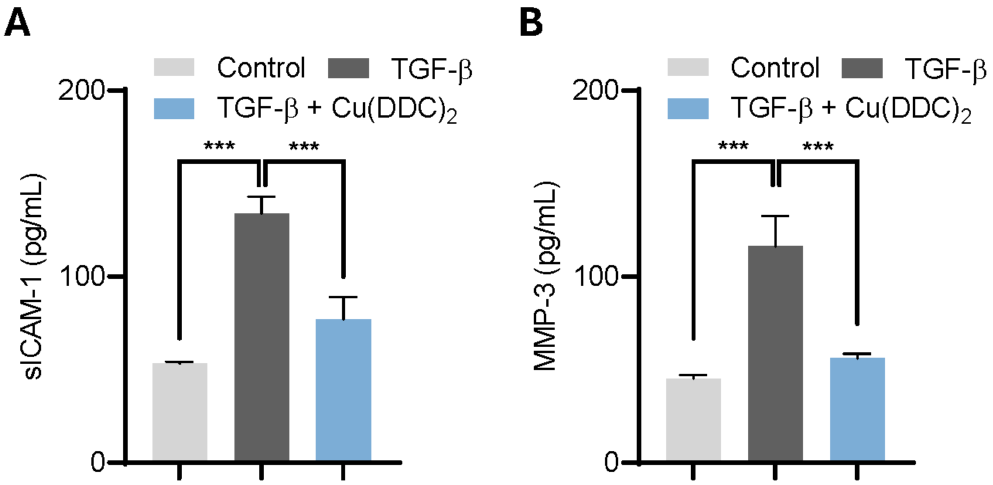

Figure S16. (A) Quantification of sICAMs by ELISA reveals a reduction following [Cu(DDC)$_2$] NP treatment. (B) MMP3 expression levels are significantly altered in response to [Cu(DDC)$_2$] NP, suggesting ECM remodeling activity. ***, P<0.001.

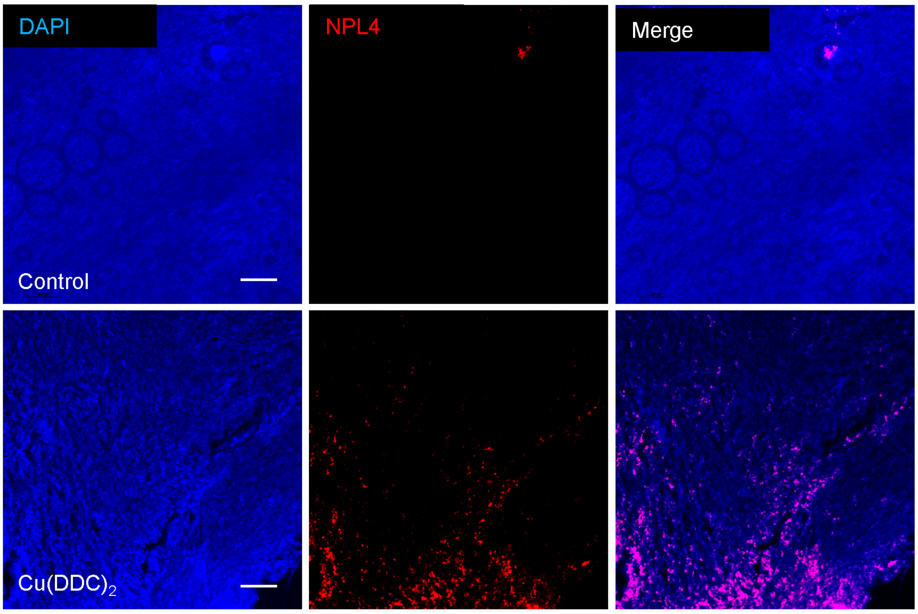

Figure S17. Immunofluorescence staining of NPL4 (red) and nuclei (DAPI, blue) in tumor sections from mice treated with PBS (control) or [Cu(DDC)$_2$] NP. Scale bar =100 μm.

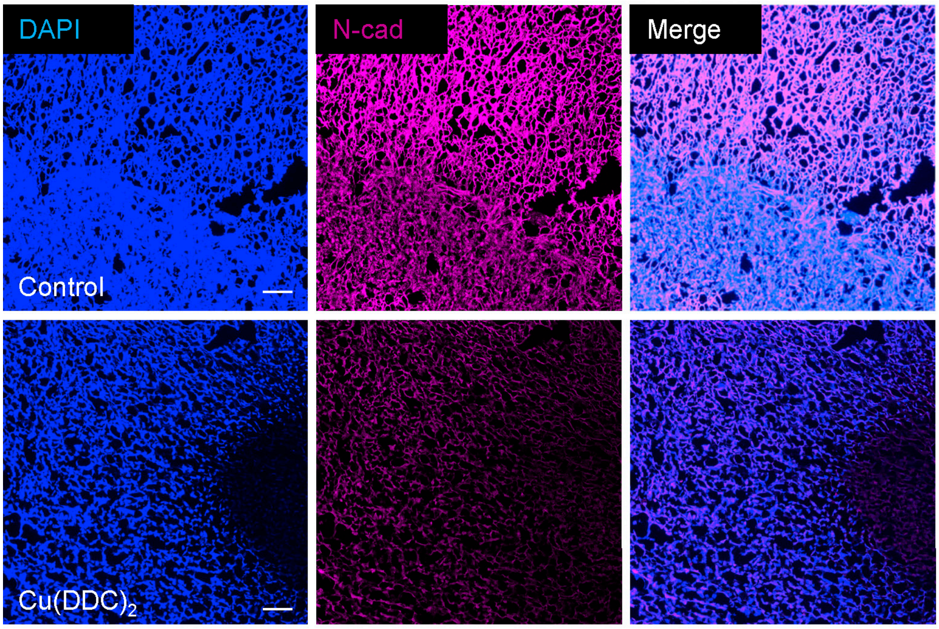

Figure S18. Immunofluorescence staining of N-cad (red) and nuclei (DAPI, blue) in tumor sections from mice treated with PBS (control) or [Cu(DDC)$_2$] NP. Scale bar =100 μm.
